# Automated measurement of lamellar thickness in human bone using polarized light microscopy

**DOI:** 10.1016/j.mex.2023.102428

**Published:** 2023-10-13

**Authors:** Nicolas Ryan, Sandra J Shefelbine, Frederic Shapiro

**Affiliations:** Department of Bioengineering, Department of Mechanical and Industrial Engineering, Northeastern University, 360 Huntington Ave, Boston, MA 02115, USA

**Keywords:** Histomorphometry, Image processing, Osteogenesis imperfecta, Automated Method for Lamellar Thickness Measurement

## Abstract

Lamellar bone formed in individuals with moderate and severe osteogenesis imperfecta (OI) is often composed of lamellae that are structurally abnormal. Measuring the thickness of these lamellae can be helpful in assessing the effect of specific collagen and collagen-related mutations on OI bone synthesis.

Manual measurement of lamellar thicknesses in large quantities is very time consuming. The method for automated measurement described in this article utilizes an image processing script to identify the average thickness of multiple lamellae automatically from histologic images of bone. This allows for faster measurements that are less prone to human error and can account for variability in the thickness of a lamella along its length.

• OI and control bone samples are prepared per the glycol methacrylate resin (JB-4 plastic) technique and viewed using polarized light microscopy.
• Ideal bone regions for measurement are identified using specific qualitative criteria designed to ensure uniform and accurate thickness measurements.
• The method was validated with dataset containing 211 lamellae from control bone and 212 lamellae from OI bone.

## Graphical abstract

**Figure:**
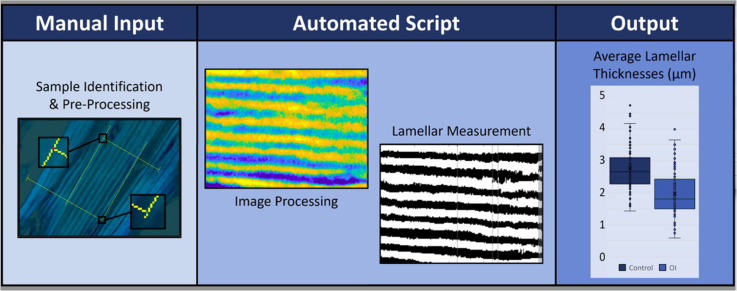

Specifications table

**Table:** 

Subject area:	Medicine and Dentistry
More specific subject area:	Lamellar Measurement in Cortical Bone
Name of your method:	Automated Method for Lamellar Thickness Measurement
Name and reference of original method:	Chow J, Ryan N, Shefelbine SJ, Shapiro F. Lamellar thickness measurements in control and osteogenesis imperfecta bone, with development of a method of automated thickness averaging to simplify quantitation. Matrix Biology 2023; 116: 85–101. https://doi.org/10.1016/j.matbio.2022.12.006
Resource availability:	**Equipment:** - Olympus BX50 photomicroscope - Olympus CMOS SC30 digital camera - Olympus U-AN360P rotatable analyzer - Olympus U-TP137 polarizing quarter wave mica plate fixed compensator
	**Software:** - cellSens (Olympus LS, Tokyo, Japan) - MATLAB (Mathworks, Natick, MA) - Paint (Microsoft, Redmond, WA)

## Method details

### Manual sample preparation

#### Preparation of tissue slides

Bone specimens were obtained from individuals (5–26 years old) undergoing orthopedic surgical deformity corrections in which the bone would have otherwise been discarded. Samples of control bone from 5 patients without underlying molecular abnormalities were collected along with samples of OI bone from 8 patients from intact lower extremity long bones during osteotomy and intramedullary rod insertion procedures.

A glycol methacrylate resin technique was used to prepare bone samples for microscopy. Specimens were fixed in 10% neutral buffered formalin for 3 weeks and then decalcified until soft in 25% formic acid. Segments with dimensions 15 mm X 7 mm X 7 mm or smaller were cut from the specimens by scalpel or razor blade along either the longitudinal or transverse cortical axes and infiltrated in glycol methacrylate (JB-4 medium, JB-4 embedding kit, Polysciences, Warrington, PA) for several weeks before being embedded in JB-4 plastic. A Microm HM 350 rotary microtome (Microm International GmbH, Walldorf, Germany) was used to section tissues at 5 µm thickness. Samples were then stained with 1% toluidine blue.

The more commonly used paraffin processing technique also allows for demonstration of bone lamellae by polarizing light microscopy. With paraffin, larger samples than those processed by the glycol methacrylate resin technique can be assessed. Fixation and decalcification are the same as for the plastic-embedded technique. After decalcification, the cortical bone is cut along either the longitudinal or transverse axes; placed in increasing concentrations of alcohol; infiltrated and embedded in paraffin; cut at 7 µm thickness; and stained either with 1% toluidine blue or hematoxylin and eosin.

#### Identification of lamellar group concentrations for measurement

**Fig. 1:**
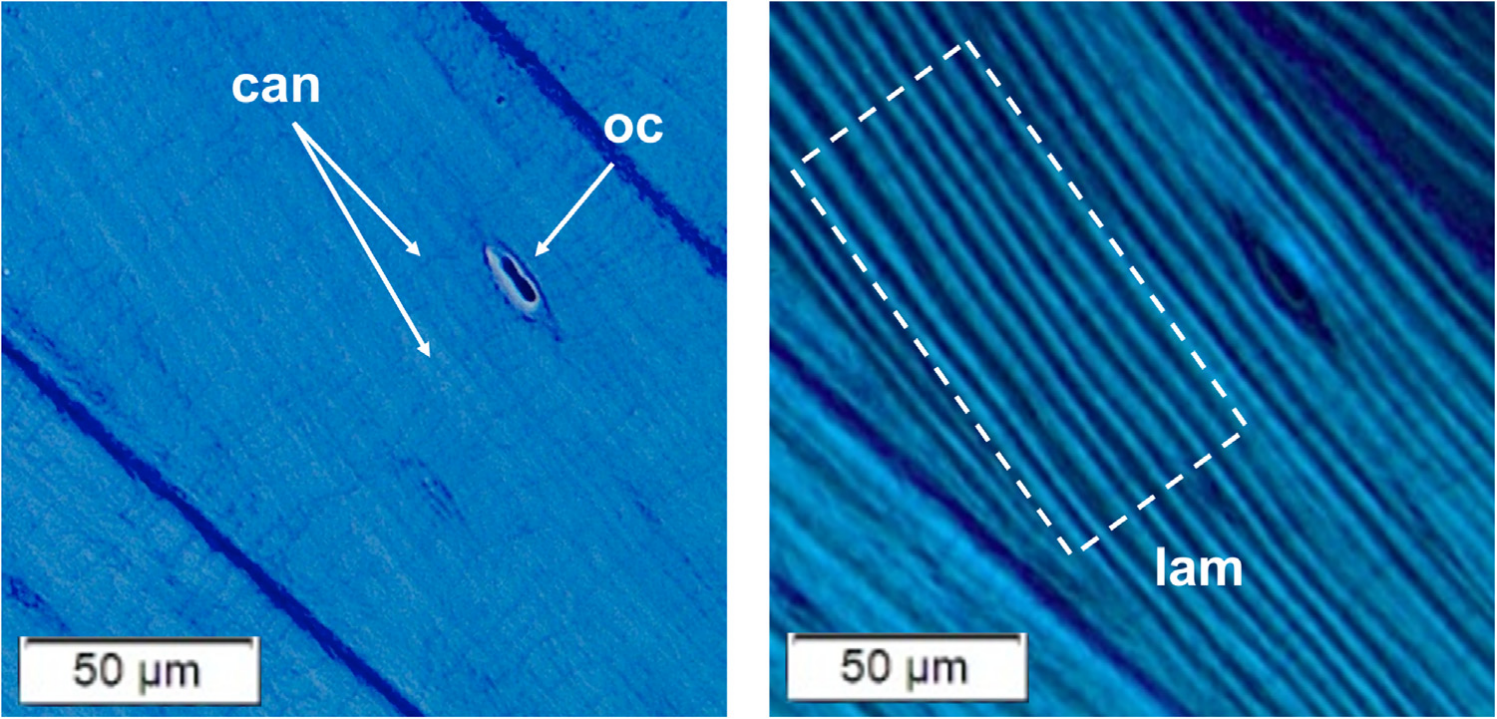
**Left:** An area of normal cortical bone from a control patient, viewed under light microscopy at 20x magnification. An elongated osteocyte (oc) and clearly visible canaliculi running perpendicular to the direction of lamellae (can) indicate that this is a high quality area for measurement. **Right:** The same area of bone at the same magnification, now viewed under polarized light microscopy. Many parallel lamellae of relatively uniform thickness are adjacent to one another (lam). This area meets the criteria for measurement.

Prepared bone slides were viewed under light microscopy and polarized light microscopy. An Olympus BX50 photomicroscope was used. The microscope was equipped with a rotatable analyzer and a γ 137 nm polarizing quarter wave mica plate fixed compensator for polarization studies. An Olympus CMOS digital camera was affixed to the microscope for digital photography. Histomorphometric measurements were taken using cellSens (Olympus LS, Tokyo, Japan) imaging software. Light microscopy was used at 4X and 10X magnification to identify high quality areas for measurement (Fig. 1). Characteristics of ideal measurement areas included the following:

- Accumulation of 16–20 lamellae adjacent to one another
- Qualitatively regular thicknesses throughout
- Mature osteocytes appearing flattened and elongated along the longitudinal axis of the lamellae
- Canaliculi passing from the walls of the osteocyte lacunae at near right angles to the lamellae
- Circular osteons cut without obliquity showing lamellae circumferentially arrayed around a central circular vascular space (in cross-sections of bone)

Areas with fewer than 16 or greater than 20 lamellae were acceptable, and the sample for this study contained several such areas. This range was recommended to keep areas approximately the same size for greater statistical accuracy. Additionally, larger areas containing greater than 20 lamellae were more likely to contain osteocytes or other irregularities that would cause the automated measurement script to discard more data. Approximately regular thicknesses across bright and dark lamellae indicated favorable sectioning in the true transverse or longitudinal plane, while a large qualitative variance in thicknesses indicated oblique cuts. Oblique cut angles could artificially alter the measured thicknesses of lamellae. Fig. 1e, f, and g in the related research article further define the characteristics of optimal measurement areas [1].

After each measurement area was chosen, magnification was increased to 20X and polarization was activated. With polarization active, lamellae appeared as alternating bright and dark bands. The microscope stand was rotated relative to the polarized light to create maximum contrast between bright and dark lamellae, and a photo of the tissue sample was captured with the digital camera.

#### Pre-processing of sample images

**Fig. 2:**
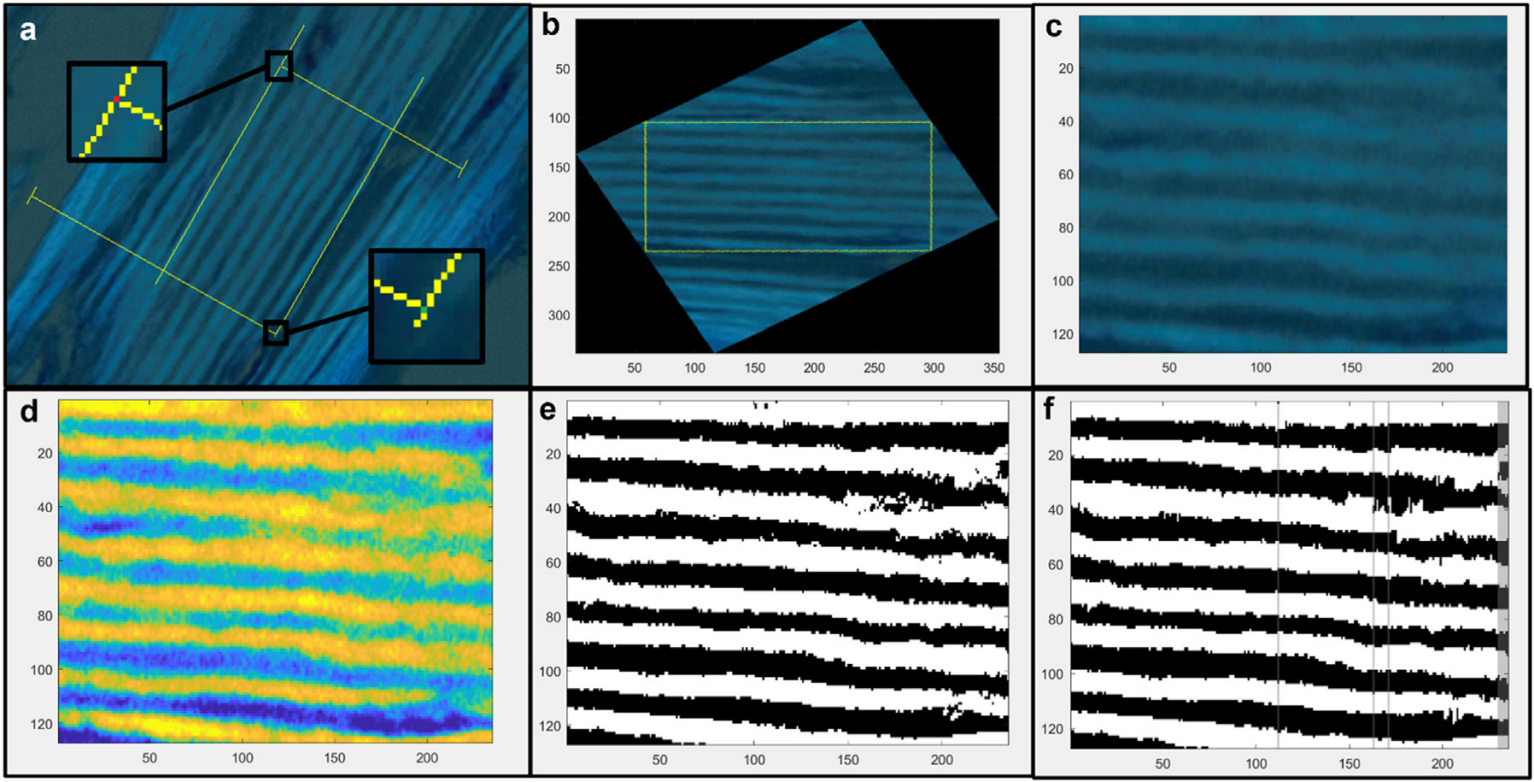
**a:** An example of a pre-processed image of a sample of control bone. The image is taken under polarized light microscopy at 20x magnification and marked for compatibility with the script. **b:** The image is rotated so that the lamellae run horizontally across the screen. **c:** The image is then cropped to include only the measurement area. **d:** A grayscale filter is applied to the image and the contrast is increased. **e:** The image is recreated as a binary measurement image with each pixel marked as either bright or dark. **f:** Columns of pixels that don't meet the criteria for acceptance are grayed out and excluded from measurement.

Once sections of a bone sample were selected for measurement and an image of each measurement area was captured at 20x magnification, markings were made manually on the digital photographs to allow for processing by the script. Using a tool provided by the cellSens imaging software, a yellow rectangle was created enclosing the extent of each measurement area. Then, the images were saved and opened for editing in Paint (Microsoft, Redmond, WA), where the corners of each rectangle were labeled to enable the script to reorient the image for measurement. Red pixels were placed at the top corners of the rectangle and green pixels were placed in the bottom corners (Fig. 2a). Once these pre-processing steps were complete, the images were viable inputs to the automated lamellar measurement script.

### Automated lamellar measurement

#### Processing of bone image

The first function of the script isolates the area of interest and re-orients it so that the lamellae run horizontally across the screen. The positions of the red markers are used to calculate the angle of rotation required. The script calculates the degree of rotation required to align the red markers horizontally and then applies that rotation to the image (Fig. 2b). The script then crops the image to remove any pixels outside of the measurement area defined by the yellow rectangle. The script locates the rows and columns of pixels that have been marked yellow and creates a new image of the area just inside these lines (Fig. 2c).

The final alteration made to the image is a contrast adjustment. The image is first converted to grayscale using MATLAB's (Mathworks, Natick, MA) ‘rgb2gray’ function. Next, the ‘imadjust’ function is used to increase the contrast in the image. This function adjusts the contrast of the image so that 1% of the data is saturated at low and high intensities. This results in an image where the bright and dark lamellae are more easily distinguishable from one another (Fig. 2d).

#### Generation of measurement image

Following adjustment of the image, the script marks each pixel as either bright or dark lamella. At the beginning of this process, a new image with the same dimensions as the measurement area is created. For each pixel in the original measurement area, the average intensity of the nearest 20 pixels in its column is calculated. If the intensity of the pixel is greater than that average, the pixel is marked as bright and its corresponding pixel in the new image will be white. Otherwise, it is marked as dark, and its corresponding pixel will be black. This results in a binary image for measurement that contains only black and white pixels (Fig. 2e).

To assist with measurement, ‘floating’ pixels, a single pixel of one color surrounded by another color, are recolored to fit the lamella they are found in. The script scans through each column in the image from top to bottom. If, within a lamella, a group of 2 or fewer pixels of the wrong color are encountered, the colors of the pixels are switched to match their surroundings. This has a very minor effect on the image overall, but greatly increases the number of columns that can be included in measurement of the image.

#### Measurement of lamellae

The script then measures the thickness of each lamella in the image, column by column. The measured thickness of each lamella in the current column is recorded as the number of consecutive bright pixels for bright lamellae or the number of consecutive dark pixels for dark lamellae. When a bright pixel follows a dark pixel, or vice versa, it indicates to the script that a new lamella is being measured. The length, in pixels, of each bright and dark section in a column is recorded and saved in matrices.

After the entire image has been counted, columns of pixels that would introduce error to the measurement are identified and removed from calculations. In the final image, these columns that have been removed are grayed out. The first criterion for removal of a column is that the first pixel in the column is the wrong color. This indicates either that the first lamella measured was cut off by the measurement window or that a lamella from outside the measurement window has been erroneously included in the image. If the majority of first pixels are bright, columns that begin with a dark pixel are removed, and vice versa.

The next criterion for removal is the identification of the wrong number of either bright or dark lamellae. Prior to running the script, the operator manually counts the number of bright and dark lamellae in the measurement area. These values are set manually before the script is activated. A column having more than the expected number of lamellae indicates that there may have been some floating pixels in the middle of a lamella that the script erroneously counted as a new lamella. A column showing less than the expected number of lamellae indicates that two lamellae may have blended together and have been counted as one, or that the column is from a part of the image where not all of the lamellae of interest are present. Columns in which the wrong numbers of lamellae were counted are removed (Fig. 2f).

The final measured thickness for each lamella is obtained by averaging its width, in pixels, across all of the columns that were not removed. This is valuable because it returns an average thickness of the lamella across the entire measurement area, rather than lamellar thickness at a single point. Thicknesses are then converted from pixels to micrometers. The length of one pixel at the magnification level used is 0.3185 µm.

### Automated measurement script validation

A dataset of 211 lamellae from subjects without OI and 212 lamellae from subjects with OI was created to validate the script. The thicknesses of these lamellae were manually measured one-by-one before being measured again using the automated script. For each lamella in the validation dataset, the percent difference between automated and manual measurements was calculated. The mean of the absolute values of these percent differences was 18.9%. The mean number of individual thickness measurements taken per lamella was 86 for normal bone and 72 for OI bone. A comprehensive assessment of the data collected during the validation of this method has been presented in the related research article [1]. In this article, Table 2 compares measurements of bright and dark lamellae using the two methods in the control group (3 patients) and OI group (5 patients). Fig. 4a and b illustrate overall thickness comparisons (4a) and bright and dark comparisons (4b) in control and OI groups using the two methods.

Paired statistical tests were used to determine whether the measurement made by the automated averaging method differed significantly from the manual measurements on the same lamellae. A Lilliefors test determined that the differences between the automated and manual measurements of the same lamellae were not normally distributed (α = 0.05, *p* < 0.005). For this reason, a non-parametric test was used. A two-sided paired sign test on the median of the differences did not show that the automated and manual measurements were significantly different (α = 0.05, *p* = 0.0518).

## Measurement protocol

**Fig. 3:**
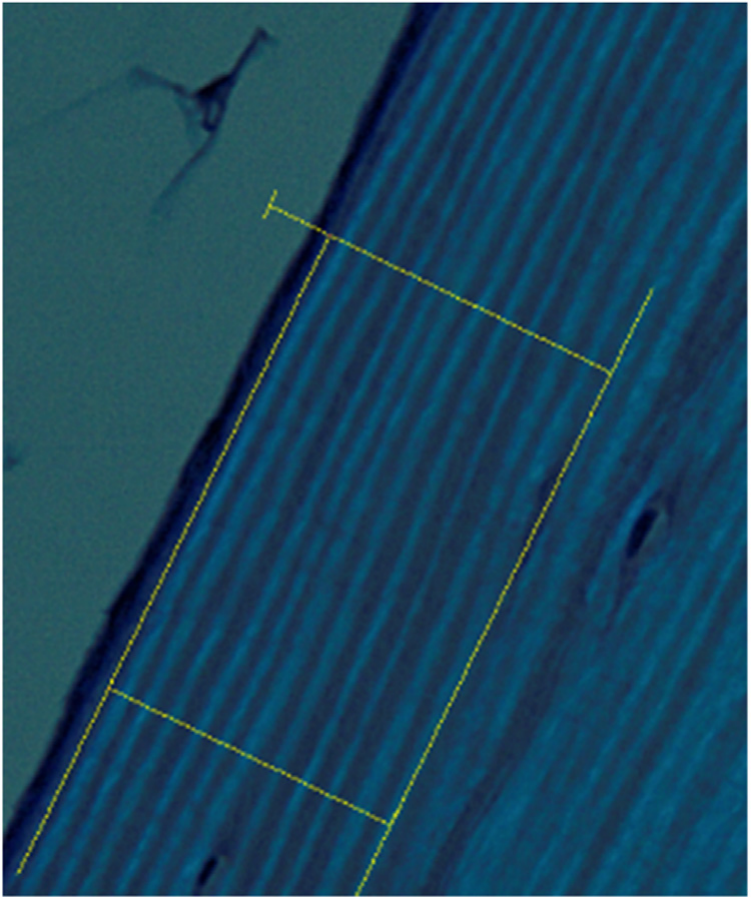
Example image for measurement protocol, step 1.2.

The following are instructions for utilizing the automated lamellar thickness measurement MATLAB script (see supplementary material). In order to create optimal input images for the program, bone sample slides should be prepared and measurement areas should be selected as described in the related research article [1]. These instructions pick up after images of stained bone samples under polarized light have been captured at 20x magnification (Fig. 3). The names and descriptions of key variables used in the automated measurement script are listed in Table 1.

1. Sample Image Pre-processing
  1.1. Open the newly acquired image in cellSens Entry. Use the ‘perpendicular lines’ tool to form two sides of a rectangle around the measurement area. Position the lines such that one is parallel to the lamellae and the other is perpendicular to them.
    1.1.1. Use the following RGB color code for these lines:

**Table 1:** The names and descriptions of key variables used in the automated measurement script. Inputs are parameters set by the user according to the properties of the sample image. Outputs are the averaged lamellar thicknesses measured from the image and the number of columns sampled to produce the measurements.

Important Variables	
Variable Name	Content
**Inputs**	
filepath	File location of the pre-processed sample image
xnBrightLamellae	Number of measurable bright lamellae in the image
xnDarkLamellae	Number of measurable dark lamellae in the image
RedColor	RGB code for the color of the red pixel markers
GreenColor	RGB code for the color of the green pixel markers
PixelLength_um	Physical length in micrometers of one pixel in the image
**Outputs**	
AvgBrightLengths_um	Average measured thickness for each bright lamella in the image, in micrometers
AvgDarkLengths_um	Average measured thickness for each dark lamella in the image, in micrometers
ngSets	Total number of measurable columns in the image

**Red:** 255

**Green:** 255

**Blue:** 0

1.2. Select the perpendicular lines, copy them, and paste the copy onto the image. Reposition these lines without rotating them to form the missing sides of the rectangle enclosing the measurement area.
1.3. Use the ‘burn in info’ tool to make these markings permanent on the image. Save the image as a .tif file and exit the program.
1.4. Open the .tif file in Microsoft Paint.
1.5. Use the pencil tool with a size of 1 pixel to recolor one pixel at the top corners of the yellow rectangle.
  1.5.1. The first top corner is the corner closest to the top of the image. The second is the other corner along the yellow line parallel to the lamellae.
  1.5.2. Use the following RGB color code for these points:

**Red:** 237

**Green:** 28

**Blue:** 36

  1.6. With the same tool, place green pixels at the other corners of the box.
    1.6.1. Use the following RGB color code for these points:

**Red:** 34

**Green:** 177

**Blue:** 76

**Fig. 4:**
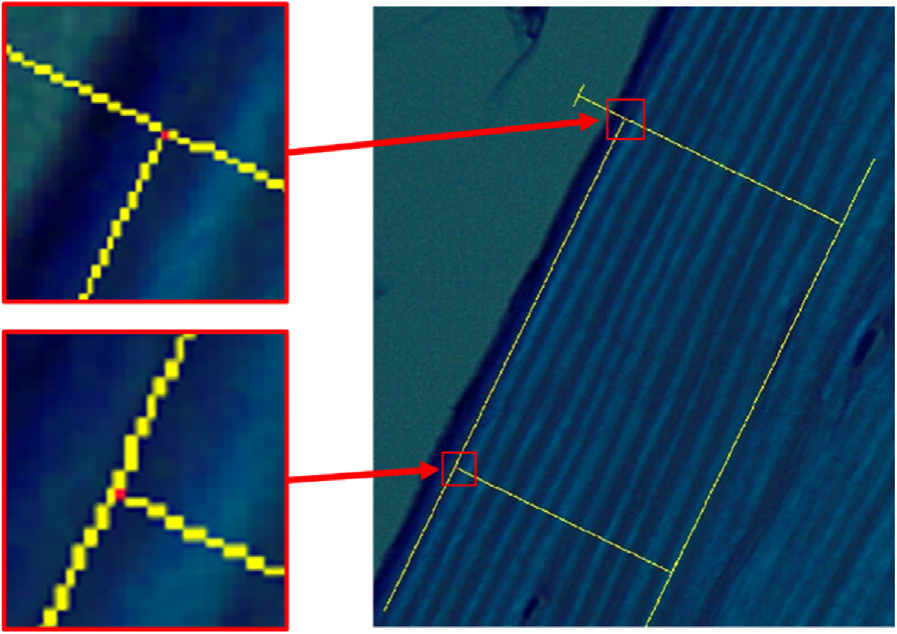
Example image for measurement protocol, step 1.5.1.
  2.4. Change the variable ‘xnDarkLamellae’ to the expected number of dark lamellae and the variable ‘xnBrightLamellae’ to the expected number of bright lamellae.
  2.5. Run the program again. Record the following variables:
    2.5.1. Average lengths in microns of all bright lamellae measured (‘AvgBrightLengths_um’)
    2.5.2. Average lengths in microns of all dark lamellae measured (‘AvgDarkLengths_um’)
    2.5.3. Total number of columns in measured image (‘nSets’)
    2.5.4. Number of columns in measured image that were not discarded due to potential measurement error (‘ngSets’)
  2.6. After the program has completed, MATLAB will generate six figures showing the processing steps of the measurement image. The sixth figure shows the final measurement image with excluded columns grayed out.

**Fig. 5:**
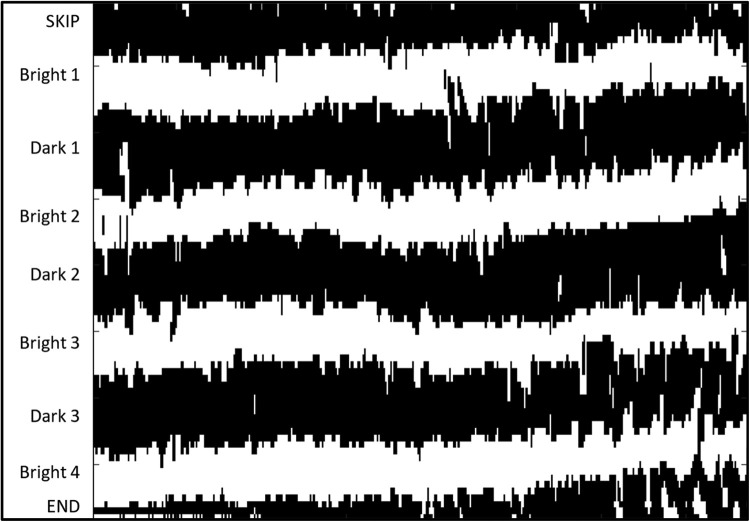
Example image for Measurement protocol, step 2.3.3.1.
      2.3.3.2. Example: Fig. 6 has 6 bright and 5 dark lamellae. Note that the lamella labeled ‘Bright 6’ is partially cut off by the frame on the left side. Columns where this is the case will not be measured.

  1.7. Save the edited image.
2. Automated Lamellar Measurement
  2.1. Open the automated measurement script in the MATLAB program editor.
  2.2. Change the variable ‘filepath’ to the location of the edited .tif file and run the program.
  2.3. Examine Fig. 4 (generated in MATLAB) and determine the number of lamellae expected to be identified by the program:
    2.3.1. Is the very top row of pixels mostly bright or mostly dark? If dark, the closest bright band to the top of the image is where measurement will start. Opposite if top row of pixels is mostly bright.
    2.3.2. Starting from the top, count the bright and dark bands running horizontally across the image.
    2.3.3. Stop counting once a band is encountered that is mostly cut off by the bottom of the frame. Bands that have small segments cut off by the frame are acceptable, as measurements in these areas will be excluded from calculation.
      2.3.3.1. Example: Fig. 5 has 4 bright and 3 dark lamellae.

**Fig. 6:**
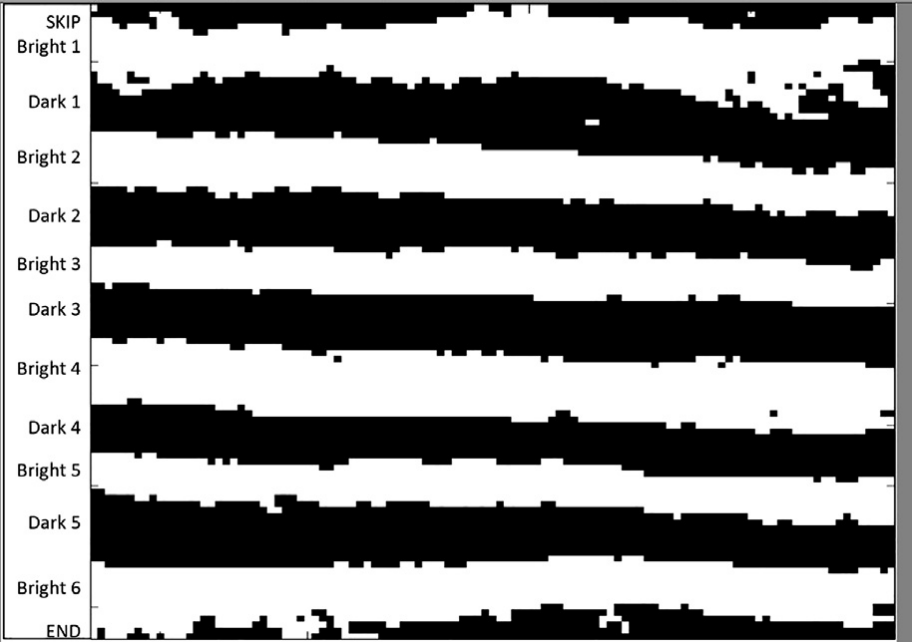
Example image for Measurement protocol, step 2.3.3.2 has 6 bright and 5 dark lamellae.

## Ethics statements

All bone specimens were obtained during operative procedures to correct bony deformity performed by the corresponding author (FS) at Boston Children's Hospital, Harvard Medical School, Boston MA, USA. The cortical bone used for this study would otherwise have been discarded. The investigation was approved by the hospital Institutional Review Board.

## CRediT authorship contribution statement

**Nicolas Ryan:** Methodology, Investigation, Software, Validation, Formal analysis, Writing – original draft, Visualization. **Sandra J Shefelbine:** Resources, Writing – review & editing, Supervision, Project administration. **Frederic Shapiro:** Conceptualization, Methodology, Investigation, Resources, Writing – review & editing, Project administration.

## Data Availability

The data that has been used is confidential.

## Appendix Supplementary materials

Image, application 1
